# Supplementary material for: Cell type-specific multi-omics analysis of cocaine use disorder in the human caudate nucleus
Source: Nat Commun. 2025 Apr 9;16:3381. doi: 10.1038/s41467-025-57339-y (PMC11982542; doi:10.1038/s41467-025-57339-y)
Supplement: Supplementary file 1 — Supplementary Information [file 41467_2025_57339_MOESM1_ESM.pdf]

Supplementary Figure 1

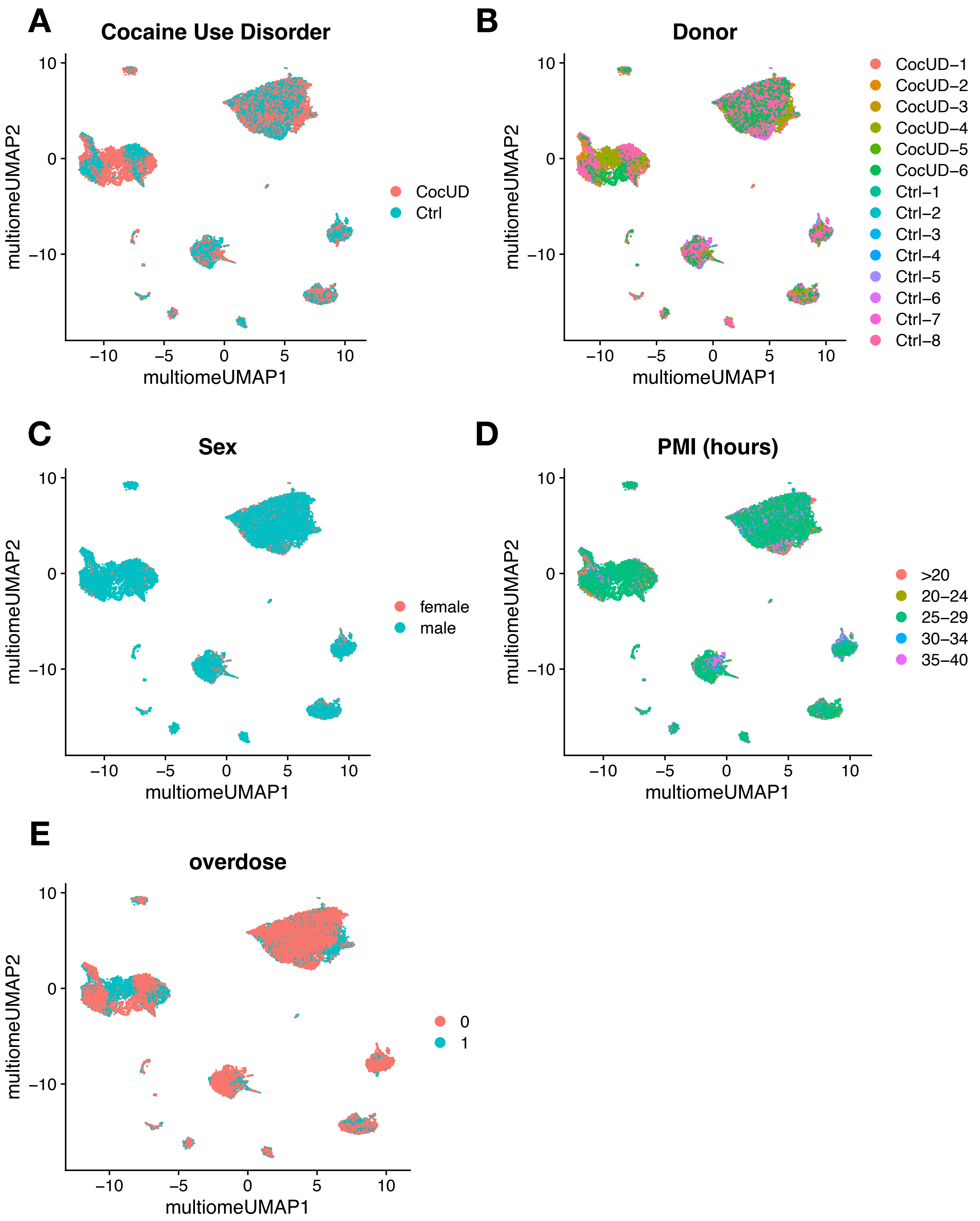

Supplementary Figure 2

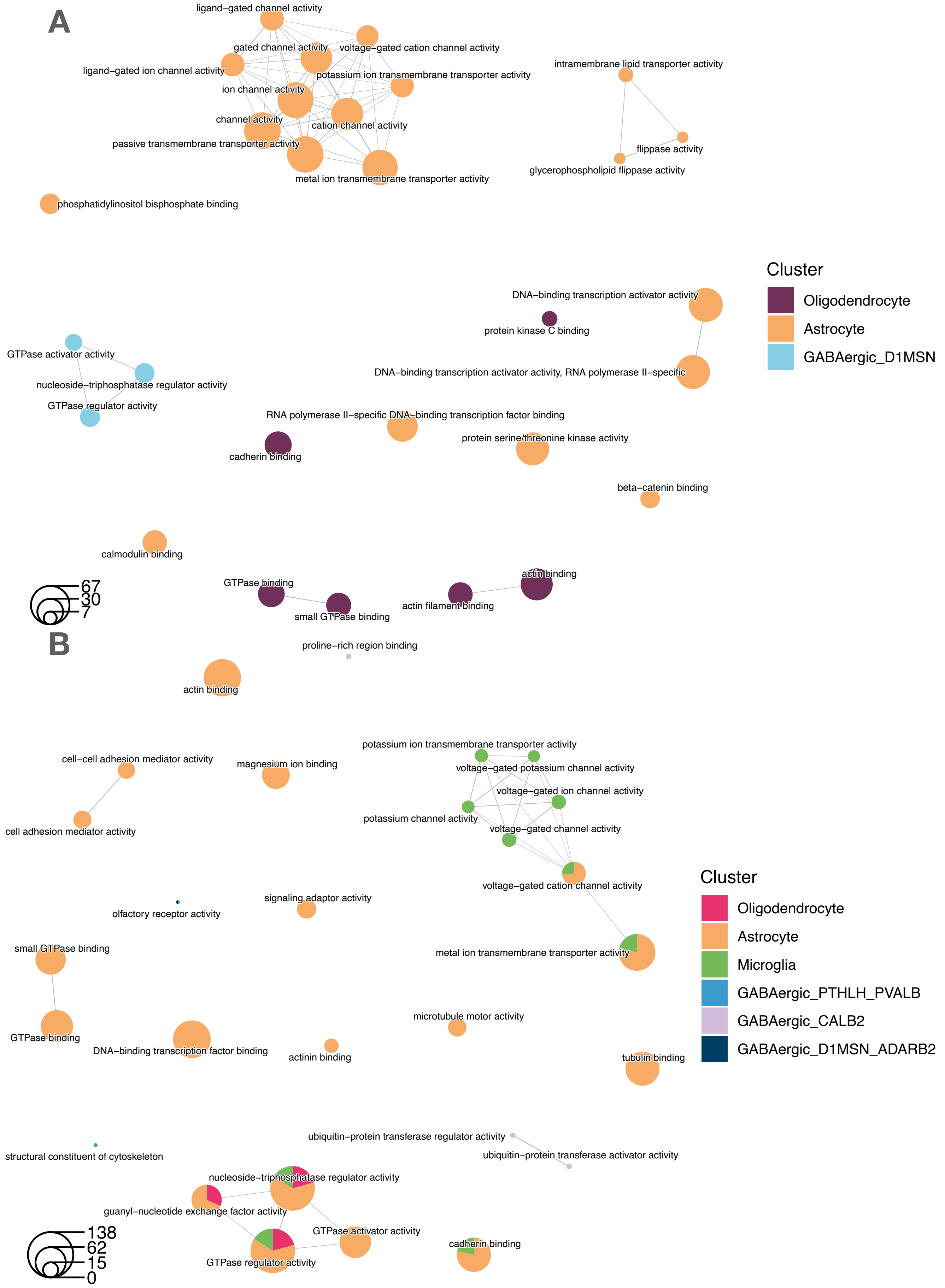

Supplementary Figure 3

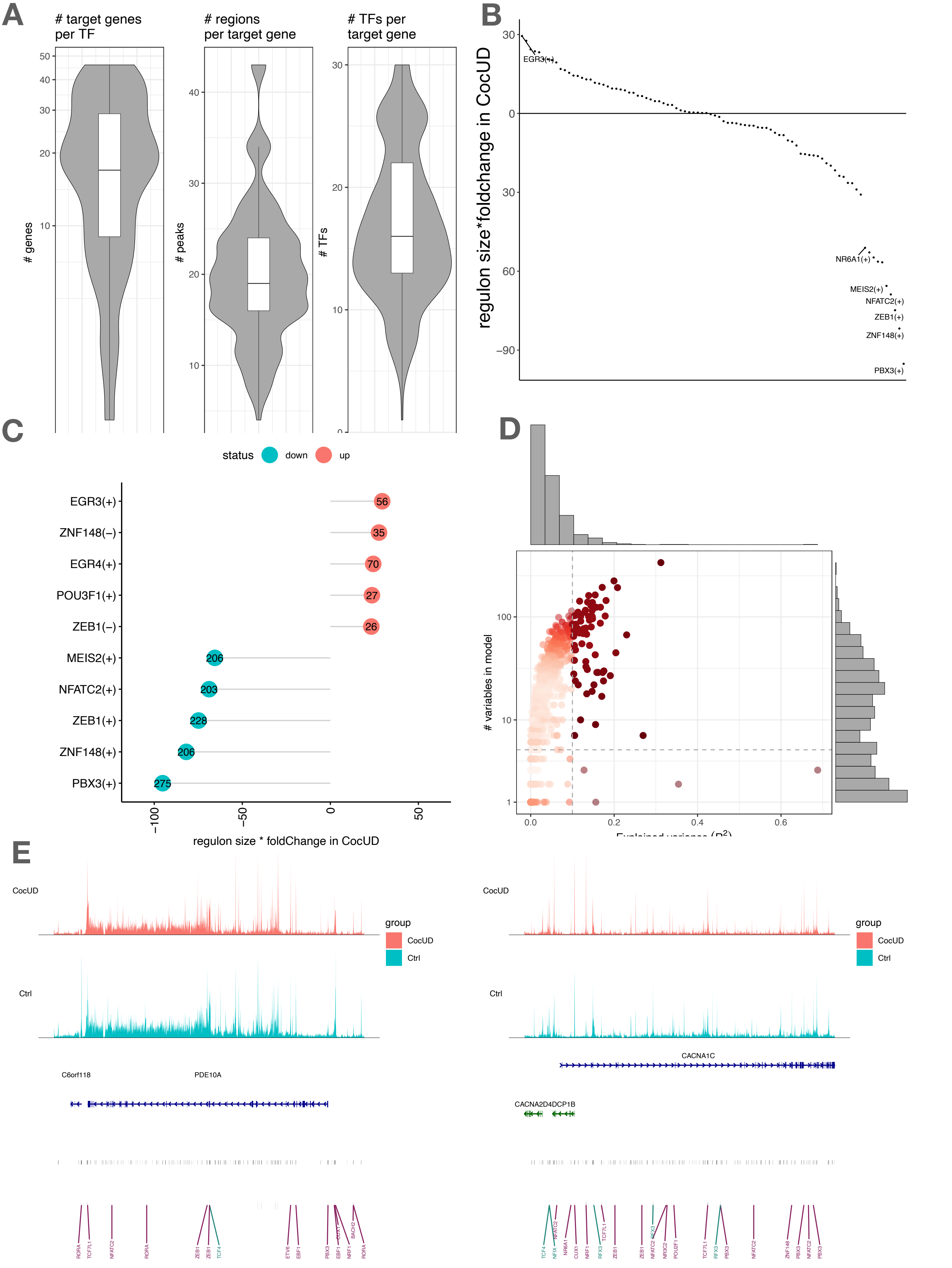

Supplementary Figure 4

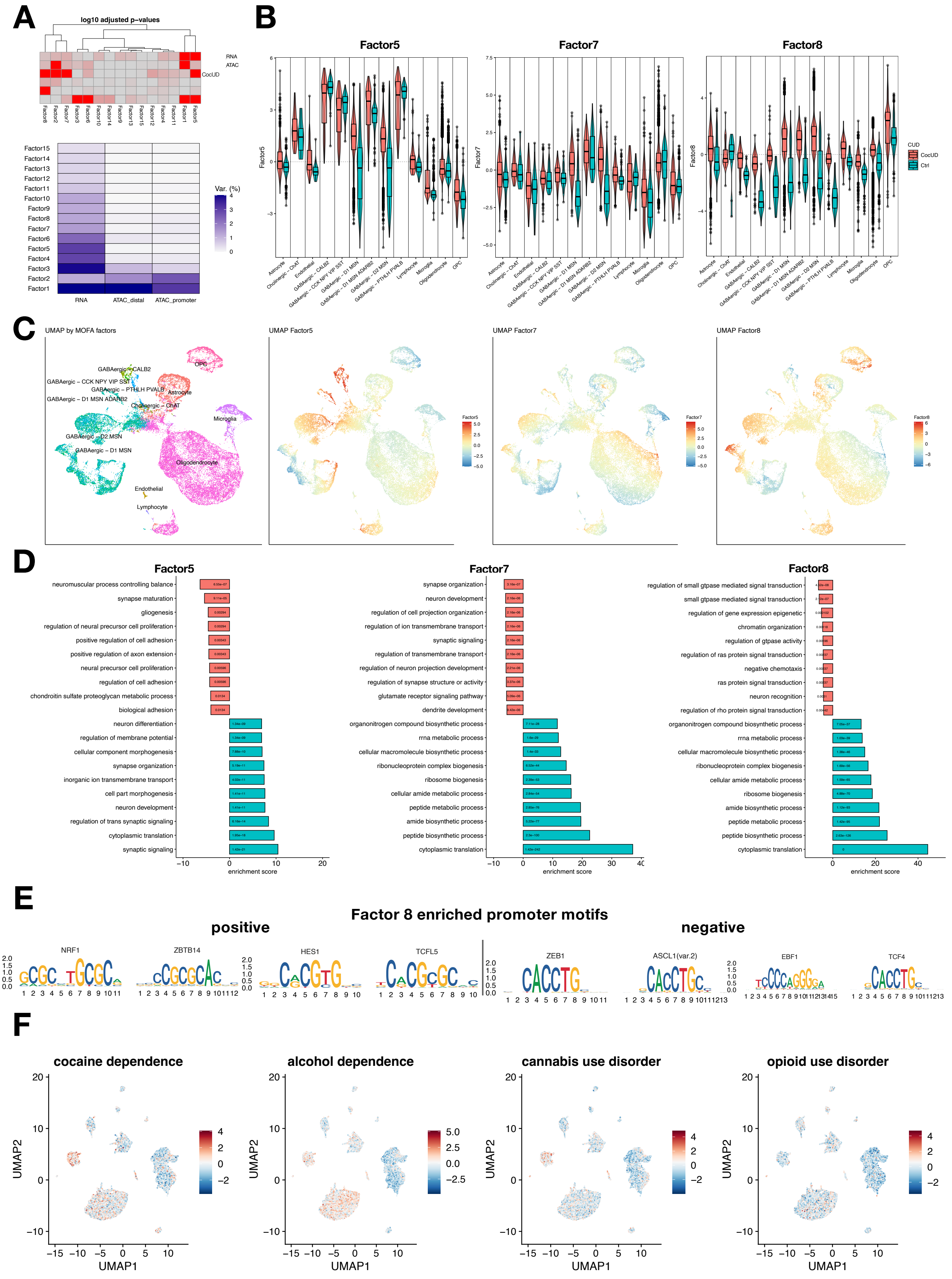

Supplementary Figure 5

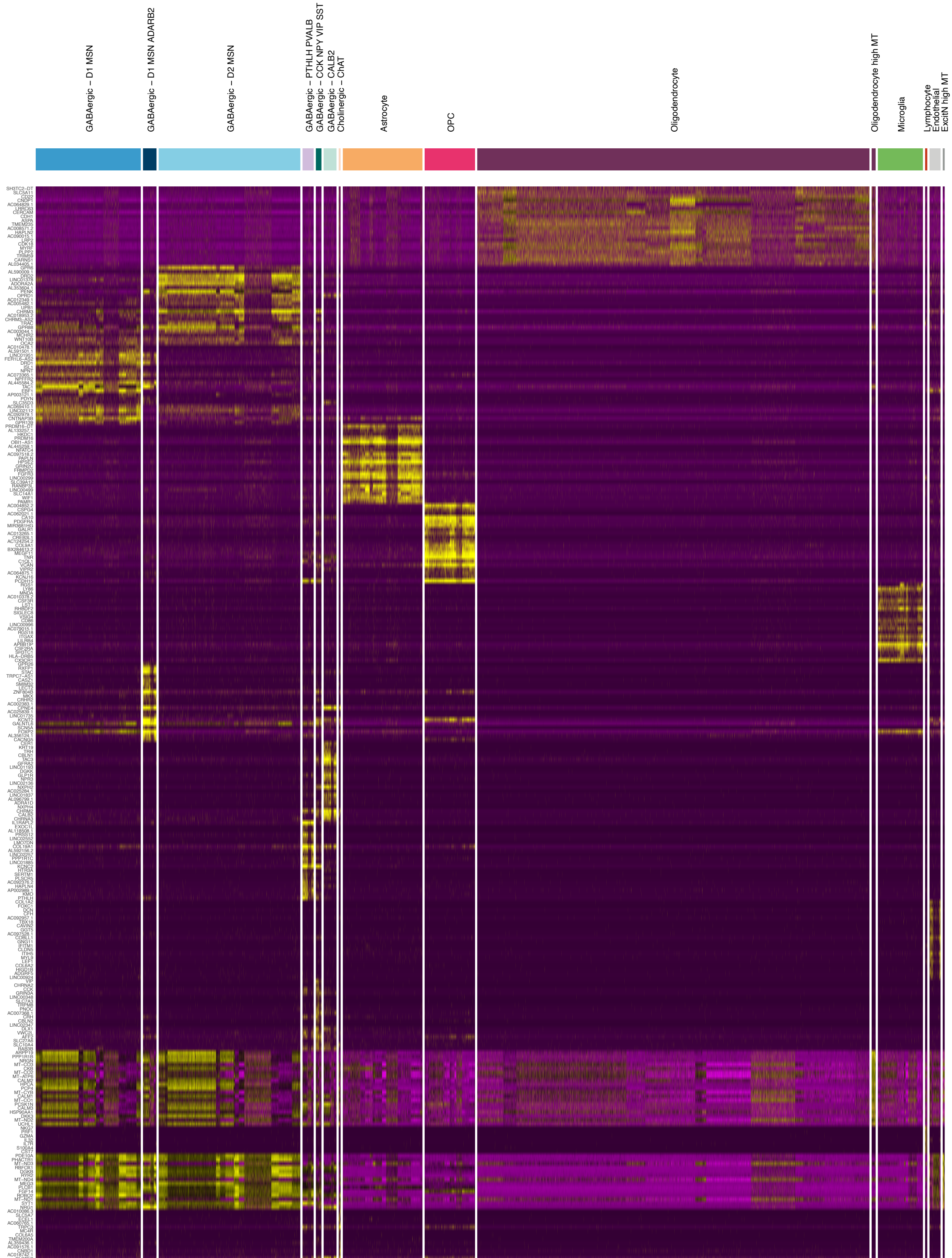

Supplementary Figure Legends

Supplementary Figure 1. Covariates by cell type. UMAPs colored by A) CocUD case-control status, B) donor, C) sex, D) PMI (hours), E) overdose. CocUD = cocaine use disorder, Ctrl = control, PMI = postmortem interval, 0 = no, 1 = yes.

Supplementary Figure 2. Gene Ontology Overrepresentation Analysis of Differentially Accessible Peaks. emapplot depicting the results of the Gene Ontology overrepresentation analysis in a cell type specific manner, for A) promoter peaks and B) distal peaks, each circle represents one GO term as a pie chart, indicating the strength of enrichment in the different cell types, represented by color. Edges indicate semantically similar GO terms, with overlapping genes; circle size indicates the number of genes in the pathway.

Supplementary Figure 3. Gene Regulatory Network of D1- and D2-MSNs, continued. A) violin plots depicting transcription factor - target gene statistics for the D1-/D2-MSN GRN derived by Pando. B) results of regulon scoring for transcription factors from the D1-/D2-MSN GRN. Regulon size was multiplied by its associated log2foldchange from the differential regulon expression analysis. C) top 5 results for upregulated (red) and downregulated (blue) regulons in CocUD from the scoring approach in the D1-/D2-MSN GRN. The regulon size is shown inside circles. D) goodness of fit indices for the D1-/D2-MSN GRN. E) coverage plot for target gene *PDE10A* indicating the TF binding sites predicted by Pando. F) coverage plot for target gene *CACNA1C* indicating TF binding sites predicted by Pando. Blue = Ctrl, red = CocUD, TF = transcription factor, GRN = gene regulatory network, MSN = medium spiny neurons.

Supplementary Figure 4. Multi-Omics Factor Analysis (MOFA) and single-cell disease risk scores. A) association of MOFA factors with CocUD, number of RNA features, number of ATAC features, postmortem interval, sex and cell types (upper) and variance explained by RNA, distal ATAC peaks and promoter ATAC peaks for each factor. B) factor loadings split by CocUD/Ctrl status for each cell type for CocUD-associated factors 5, 7, and 8. C) MOFA UMAP projection colored by cell types (left), factor5 loadings in MOFA UMAP (middle left), factor7 loadings in MOFA UMAP (middle right), factor8 loadings in MOFA UMAP (right). D) results from gene-set enrichment analysis of factor5 (left), factor7 (middle), and factor8 (right). E) enriched promoter motifs of factor8 shown for positive (left) and negative loadings (right). F) projection of scDRS disease scores of cocaine dependence (Gelernter et al., 2014), alcohol dependence (Zhou et al., 2020), cannabis use disorder (Johnson et al., 2021), opioid use disorder (Polimanti et al., 2020) on the UMAP of cell types in the caudate nucleus. MOFA = Multi-Omics Factor Analysis, CocUD = cocaine use disorder, nFeatureRNA = number of RNA features, nFeature ATAC = number of ATAC features, PMI = postmortem interval.

Supplementary Figure 5. Cluster annotation. Heatmap depicting the highly expressed genes in each cell type cluster compared to gene expression in all other cell type clusters. Yellow = high expression, pink = low = expression.
