# Supplementary material for: Cell type-specific multi-omics analysis of cocaine use disorder in the human caudate nucleus
Source: Nat Commun. 2025 Apr 9;16:3381. doi: 10.1038/s41467-025-57339-y (PMC11982542; doi:10.1038/s41467-025-57339-y)
Supplement: Supplementary file 2 — Description of Additional Supplementary Files [file 41467_2025_57339_MOESM2_ESM.docx]

**Inventory of Supplementary Information - Cell type-specific Multi-Omics Analysis of Cocaine Use Disorder in the Human Caudate Nucleus**

Supplementary Data – S1: Phenotypic information and quality

Table listing age, sex, postmortem interval (in hours), drugs at time of death, overdose, and quality criteria of single-nuclei preprocessing

Supplementary Data – S2:

Differentially Expressed Genes identified by a two-sided Wilcoxon rank sum test at nominal significance (p<0.05) in all major cell types, with Bonferroni adjustment for multiple testing (P adj).

Supplementary Data – S3:

Differentially Accessible Peaks identified by a two-sided Wilcoxon rank sum test at nominal significance (p<0.05) in all major cell types, with Bonferroni adjustment for multiple testing (P adj).

Supplementary Data – S4:

Bonferroni-corrected p-values from two-sided chi-square test to test overrepresentation of differentially accessible peaks compared to the genomic background (Figure 2H).

Supplementary Data – S5:

Differentially Accessible Motifs identified by a two-sided Wilcoxon rank sum test at nominal significance (p<0.05) in all major cell types, with Bonferroni adjustment for multiple testing (P adj).

Supplementary Data – S6:

Connections in Gene Regulatory Network of D1/D2 MSNs, identified using regression models as implemented in pando (Fleck et al., 2022) and FDR adjustment for multiple testing (padj).

Supplementary Data – S7:

Regulons of D1- and D2- MSNs, identified using regression models as implemented in pando (Fleck et al., 2022) and FDR adjustment for multiple testing (padj).

Supplementary Data – S8:

Druggability Analysis for highly connected target genes and log2FC from DE analysis in D1- & D2-MSNs.

Supplementary Data – S9:

Results from Gene Ontology overrepresentation analysis based on the cell type-specific differential expression analysis (Table S3), using a Fisher's exact test and Bonferroni correction as implemented in clusterProfiler.

Supplementary Data – S10:

scDRS results for cocaine dependence CocUD, alcohol use disorder (AUD), cannabis use disorder (CanUD), and opioid use disorder (OUD).
